# Does the ThinPrep Imaging System increase the detection of high-risk HPV-positive ASC-US and AGUS? The Women and Infants Hospital experience with over 200,000 cervical cytology cases

**DOI:** 10.4103/1742-6413.54917

**Published:** 2009-08-06

**Authors:** M. Rudhul Quddus, Theresa Neves, Mary E. Reilly, Margaret M. Steinhoff, C. James Sung

**Affiliations:** Department of Pathology, Women and Infants Hospital and the Warren Alpert Medical School of Brown University, Providence, RI, USA

**Keywords:** ASC-US, AGUS, TIS

## Abstract

**Background:**

Published reports have demonstrated that introduction of the ThinPrep Imaging System (Imager) to the cytology screening services has increased the detection rate of high-grade squamous intraepithelial lesions (HSILs). In accordance with recent clinical treatment guidelines, patients with atypical squamous or glandular cells of undetermined significance (ASC-US or AGUS) are often tested for high-risk HPV infection using the Hybrid Capture HPV DNA test. We took the opportunity to investigate whether the Imager had resulted in any significant differences in our diagnostic categories, as well as whether the Imager increased the detection of high-risk HPV-DNA-positive (HRHPV+) ASC-US or AGUS.

**Materials and Methods:**

Cytology cases with the diagnosis of ASC-US and AGUS were retrieved from the archival files of our institution during periods of 11 months prior to and 11 months after the introduction of the Imager. The total number of cases in each category was correlated with results of reflex high-risk HPV DNA testing when the latter were available. All AGUS diagnoses were correlated with subsequent biopsy follow-up. Statistical analyses were performed using the chi-Square test with Yate's Correction and Fisher's Exact test.

**Results:**

A total of 108,371 and 104,555 of ThinPrep® Pap Test (TPPT) cases were reviewed during 11 months pre- and post-imager introduction. The ASC-US rate was 5.4% in the pre-Imager and 5.3% in the post-Imager period. The HPV reflex test was 38% and 34% positive respectively in the pre- and post-Imager period (*P*>0.124). Similarly, 0.14% and 0.12% AGUS were found in the pre- and post-Imager period. The positive HPV reflex test was 14% versus 23% (*P* = 0.1690). The abnormal biopsy follow-up rate in the AGUS category was increased from 20.9% in the pre-Imager period to 31% in the post-Imager period (*P* = 0.1471). The ASCUS/SIL ratios were 1.9 and 1.6 respectively.

**Conclusions:**

The ASC-US and AGUS rates did not change statistically before and after the introduction of the Imager in our cytology laboratory. Although use of the Imager did not increase detection of HPV+ ASC-US, it did appear to increase the detection rate of HPV+ AGUS and subsequent abnormal biopsy follow-up rates in all categories. However, the increase in the detection rate did not reach the point of statistical significance

## INTRODUCTION

Detection of atypical squamous cells of undetermined significance (ASC-US) is by far the most common abnormal cervicovaginal Pap test interpretation in any cytology laboratory. Recently published reports have demonstrated that the introduction of the ThinPrep Imaging System (Imager) to the cytology screening services has increased the detection rate of high-grade squamous intraepithelial lesions (HSILs) and low-grade lesions (LSILs).[1–5] Lozano[4] reported that the ThinPrep® Imager System (TIS) has increased the detection of HSILs by 38%, LSILs by 46%, and ASC-US by 46% compared to manual screening. The ASC:SIL ratio during the study period did not change. Similarly, Papillo *et al*. reported an increase in ASC-US, atypical sqinamous cells cannot exclude HSIL (ASC-H), and LSIL rates by 34, 48 and 29% after the implementation of ThinPrep Imager.[5] Use of the TIS has led to increased detection of ASC-H as reported in multiple studies.[6,7] However, other studies have found that the sensitivity and specificity of the imager technology are equivalent to those of manual primary screening.[8] The authors however, recommended the use of automated imager for rapid screening of negative cases allowing for an increased productivity of the laboratory. The reports on ASC-US are somewhat less clear. Laboratories have observed no appreciable change in ASC-US rates or follow-up reflex HPV test results.[9] Other Laboratories have reported an increase in the ASC-US rate of detection,[10] while another study reported a decrease.[11] Report on the impact of TIS in detecting glandular lesions is relatively sparse. A study published in 2008 by Friedlander has shown that TIS is effective in identifying atypical glandular cells.[12] ThinPrep® Imaging System (TIS) (HOLOGIC, Marlborough, MA) was approved by the US Food and Drug Administration (FDA) in June of 2003. The clinical trial carried out between December 2000 and November 2001 prior to approval demonstrated that the efficiency of the instrument was at least equivalent to the manual screening in all Bethesda System recommended categories.[13] The TIS is a widely used system in the country, and we implemented the system in our high-volume academic medical center laboratory in May 2005. In accordance with recent clinical treatment guidelines, patients with atypical squamous or glandular cells of undetermined significance (ASC-US or AGUS) are often tested for high-risk HPV infection using the Hybrid Capture HPV DNA test.[14] Our high-volume cytology laboratory, which serves a relatively stable population of patients with excellent clinical follow-up, introduced the Imager approximately 2 years ago. Our pathology and cytotechnology staff has undergone no major change in personnel, providing relatively stable continuity in diagnostic criteria, and our clinicians follow standard guidelines for reflex HPV testing. So far, the performance of TIS has been evaluated only in a very few high-volume laboratories of an academic medical center.[3] Therefore, we took the opportunity to investigate whether the Imager had resulted in any significant differences in our diagnostic categories, as well as whether the Imager increased the detection of high-risk HPV-DNA-positive (HRHPV+) ASC-US or AGUS.

## MATERIALS AND METHODS

The Cytology Laboratory of Women and Infants Hospital processes over 100,000 cases a year. Our laboratory introduced ThinPrep® Pap Test (TPPT) in late 90s, and since then almost 100% of our Pap tests are ThinPrep. Only a rare conventional smear is occasionally encountered here. The ThinPrep 3000 Processor (HOLOGIC) instrument is used to prepare ThinPrep Slides following manufacturer's instructions. TIS was introduced in our Laboratory on May 01, 2005 and since then 100% cases are screened by TIS.

The cytotechnologists, cytopathologists, and the patient population did not change during the study period. We did not open up any new account with any large physician group, and no previous large physician group disaffiliated its practice with our laboratory during the entire time period of study.

Cytology cases with the diagnosis of ASC (ASC-US) and AGUS were retrieved from the archival files of our institution during periods of 11 months prior to and 11 months after the introduction of the Imager. All AGUS diagnoses were correlated with the subsequent follow-up. All cytology materials were examined by the same staff of cytopathologists and cytotechnologists during the study period. The total number of cases in each category was correlated with results of reflex high-risk HPV DNA testing when the latter were available. Reflex HPV testing was performed by using Hybrid Capture 2 method (Digene, Gaithesburg, MD) using a cocktail of probes for all 13 high-risk HPV viruses. The test was done by the Microbiology Department following the manufacturer's guidelines. Statistical analyses were performed using the chi-square test with Yate's Correction and Fisher's Exact test.

## RESULTS

A total of 108,371 cases were reviewed during the 11-month period of manual screening of ThinPrep Pap smear prior to the introduction of TIS. A total of 104,555 cases were screened in the following 11 months after implementation of the TIS.

Of these cases, during the manual screening period of 11 months, 5,884 (5.4%) cases were reported as ASC-US. HPV DNA test was performed on 5,536 cases (94%). In comparison, during the 11 - month period after the TIS introduction, 5,559 (5.3%) cases were reported as ASC-US, and HPV DNA test was done on 5,515 (99%) cases.

The rate of high-risk HPV positivity detected in ASC-US was 38% versus 34% pre- and post-TIS introduction in our laboratory. The detection rate of high-risk HPV pre- and post-TIS was not significantly different (*P* = 0.124).

In contrast, 158 (0.14%) cases of AGUS were reported during the pre-TIS and 126 (0.12%) during the post-TIS period. Of these, HPV DNA test was performed on 116 (73%) during the pre-TIS and on 102 (81%) during the post-TIS period. The high-risk detection rate pre- and post-TIS was 14% versus 23% respectively. The rate of increased detection of HPV-positive AGUS during post-TIS period was higher in our laboratory, although not statistically significant (*P* = 0.1690).

Our rate of HSILs during same pre- and post-TIS periods were 249 (0.23%) versus 260 (0.25%). The difference was statistically not significant (*P*>0.05). The ASCUS/SIL ratio was 1.9 and 1.6 respectively during the pre- and post-TIS period. Table 1 outlines the findings and statistical analysis. The cases were followed for period of 4–53 months (median 42 months for the pre-Imager group and 35 months for the post-Imager group). The follow-up data and statistical analysis are presented in Table 2.

**Table 1:** Summary of findings

		*Pre-imager*	*Post-imager*	*P-value*
Total cases		108,371	104,555	
No. of ASC-US (%)		5,884 (5.4)	5,559 (5.3)	0.2789
	Reflex HPV			
	DNA testing	5,536	5,515	0.1025
	HPV+	38%	34%	0.124
No. of AGUS (%)		158 (0.14)	126 (0.12)	
	Reflex				
	HPVDNA testing	116	102	0.6527
	HPV+	14%	23%	0.1690
ASCUS/SIL ratio		1.9	1.6	

Chi-square test with Yate's Correction

**Table 2:** Follow-up data

	*Pre-imager*	*Post-imager*	*P-value*
TNo. of AGUS	158	126	
Abnormal follow-up Bx	33 (20.9)	39 (31.0)	0.1471
LSIL	12 (7.6)	18 (14.3)	0.6645
HSIL	8 (5.1)	7 (5.6)	0.7771
Adenocarcinoma in-situ (AIS)	4 (2.5)	4 (3.2)	1.000
Invasive cervical adenocarcinoma	1 (0.6)	2 (1.6)	1.000
Endometrial adenocarcinoma	8 (5.1)	8 (6.4)	0.7884
Benign follow-up Bx	114 (72.1)	76 (60.3)	0.8938
No follow-up	11 (7.0)	11 (8.7)	0.6601

Fisher's Exact test; Figures in parentheses are in percentages

## DISCUSSION

Much has been changed in cervical cancer screening since its first introduction in 1946, mostly in last 12 years. The FDA approved the liquid-based screening in 1996, and then TIS was approved by FDA in 2003. Recently, the “2006 Consensus guidelines for the management of women with abnormal cervical cancer screening tests” were published in 2007.[14]

Biscotti *et al*.[13] reported that the sensitivity of the Imager-assisted screening system equals or exceeds the sensitivity of manual primary screening without a decreasing specificity. The comparison of high-risk HPV-positive ASC-US before and after the introduction of TIS in our laboratory shows that the change is not statistically significant. The ASC-US:SIL ratio for this time during the study period also did not change significantly. Our findings support Thrall *et al*.[9] who did not find an increase in the ASC-US detection rate after using TIS.

Compared to cervical squamous lesions, reports on the impact of TIS in detecting glandular lesions are relatively fewer. The published reports are mainly in the form of abstracts.[15,16] A recent study published in 2008 by Friedlander has shown that TIS is effective in identifying atypical glandular lesions.[12]

In the current study, the findings on AGUS appear similar to what Friedlander *et al*. has reported.[12] TIS did pick up higher number of high-risk HPV positive atypical glandular cells (AGUS) compared to the period of manual screening in the present series. However, the changes are not statistically significant (*P* = 0.1690)

The abnormal biopsy follow-up rate in the AGUS category was increased from 20.9% in the pre-Imager period to 31% in the post-Imager period. Although a percentage-wise increase in detection is seen in all categories, a significant increase is seen in the LSIL category (from 7.6 to 14.3%). As expected, the benign follow-up percentage, in the AGUS diagnosis category, is decreased (60.3 from 72.1%) in the post-Imager period compared to the pre-Imager period respectively.

Since our laboratory serves a relatively stable population and the laboratory personnel (cytotechnologists and pathologists) were the same during the study period, we can conclude that the increased detection rate of high-risk HPV-positive AGUS is authentic and can be attributed to TIS.

It is often stated that comparison of cervical cancer screening methodologies should include the detection rate of HSILs. Our rate of HSILs during same pre- and post-TIS periods were 249 (0.23%) versus 260 (0.25%). The difference was statistically not significant (*P*>0.05).

## CONCLUSIONS

The ASC-US and AGUS rates did not change statistically before and after the introduction of the Imager in our cytology laboratory. Although use of the Imager did not increase the detection of HPV+ ASC-US, it did appear to increase the detection rate of HPV+ AGUS. The biopsy follow-up of AGUS also confirms an increased detection of all category lesions by TIS. The current study appears to show that when AGUS is detected by TIS, an improvement in all categories of disease detection is likely. However, the increase in the detection rate is not statistically significantly different from the pre-Imager period yet. The full benefits of standardizing screening with imaging may be difficult to measure and quantify, since women with negative screening tests are usually not biopsied and most screening studies are therefore subject to the limitation of verification bias.

## COMPETING INTEREST STATEMENT BY ALL AUTHORS

No competing interest to declare by any of the authors.

## AUTHORSHIP STATEMENT BY ALL AUTHORS

All authors of this article declare that we qualify for authorship as defined by ICMJE http://www.icmje.org/#author.

Each author has participated sufficiently in the work and take public responsibility for appropriate portions of the content of this article.

Each author acknowledges that this final version was read and approved.

## ETHICS STATEMENT BY ALL AUTHORS

This study was conducted with approval from Institutional Review Board (IRB) (or its equivalent) of all the institutions associated with this study. Authors take responsibility to maintain relevant documentation in this respect.
